# Toward precision medicine in myotonic syndromes

**DOI:** 10.18632/oncotarget.15263

**Published:** 2017-02-10

**Authors:** Michela De Bellis, Diana Conte Camerino, Jean-François Desaphy

**Affiliations:** Department of Biomedical Sciences and Human Oncology, University of Bari Aldo Moro, Bari, Italy

**Keywords:** rare disease, myotonia, sodium channels, mexiletine, precision medicine, Neuroscience

The large number of physiological processes regulated by voltage-gated sodium channels (Na_v_) and their role in many diseases make these channels highly interesting as targets for new drugs. Current research in the pharmaceutical industry mainly focuses on identifying sodium channel blockers that may have therapeutic application in widespread pathological conditions, including epilepsy, cardiac arrhythmias, and chronic pain. There is much less research regarding sodium channel drugs relevant to rare human genetic diseases, including nondystrophic myotonias (NDM). Myotonia is a skeletal muscle condition characterized by over-excitability of sarcolemma, resulting in delayed relaxation after contraction and muscle stiffness. Inherited NDM are caused by gain-of-function mutations in hNa_v_1.4 sodium channel or loss-of-function of hClC-1 chloride channel. Severity of symptoms ranges from mild to severe, the later condition affecting daily movements and deteriorating quality of life. More recently, life-threatening events have been reported in neonates suffering from severe episodic neonatal laryngospasms caused by Na_v_1.4 mutations. After two decades of off-label use in NDM, mexiletine was recently appointed as an orphan drug in myotonic syndromes. By blocking skeletal muscle sodium channels, mexiletine can counteract sarcolemma over-excitability and alleviate symptoms, whatever myotonia is caused by sodium or chloride channel mutations. However, some myotonic patients cannot take mexiletine because of side effects or contraindications, while others, estimated to be nearly 20%, do not obtain satisfactory response to mexiletine [1]. Alternative drugs to mexiletine are definitely needed.

We are currently pursuing various strategies to identify new promising therapies for NDM. We combined electrophysiology and molecular modeling to study Na_v_ blockers, and we recently developed a pharmacologically-induced model of myotonia in the rat. First, we compared a number of marketed sodium channel blockers to mexiletine [2]. We found that the anti-myotonic activity of drugs *in vivo* was closely parallel to inhibition *in vitro* of sodium currents elicited by high-frequency voltage-clamp protocols in mammalian cells transfected with hNa_v_1.4 cDNA. Riluzole, currently indicated for amyotrophic lateral sclerosis, appeared as a very potent antimyotonic drug in the rat model and a human pilot study has been recently launched for NDM.

Second, for many years we have performed a series of structural-activity relationship studies to define the molecular interactions of mexiletine and its relative, tocainide, with Na_v_1.4 channels [3, 4]. These studies provided new insights into the relative role of pharmacophores for binding to crucial amino acid residues in Na_v_ and suggest novel lead molecules for enhancing potency, use-dependence, and stereoselective behavior. These studies eventually result in the chemical optimization of a tocainide derivative, namely To042, exerting a very potent and use-dependent inhibition of Na_v_1.4 channels [5]. Use-dependence is a cardinal characteristic of sodium channel blockers, allowing the drug to block channels in over-excited membranes that fire action potentials at high frequency, while preserving healthy tissue function. The key structural features of To042 with respect to the parent compound is the elongation of the alkyl chain and the introduction of a bulky, hydrophobic naphthyl ring close to the protonable amine. These findings further corroborate the role of structural modifications resulting in a stronger interaction with hydrophobic pockets at the channel level. Compared to mexiletine, To042 was 120 times more potent in blocking hNa_v_1.4 channels in a myotonia-like cellular condition, and 100 times more potent in improving muscle stiffness *in vivo* in the rat model of myotonia [6]. In addition, similar concentrations of To042 did not affect hERG potassium currents and exerted little cytotoxic effects *in vitro* on murine muscle cells, suggesting a satisfactory safety profile. Additional preclinical studies are warranted before to test To042 in humans.

The third strategy consists in the development of a pharmacogenetics approach. Years ago, we wondered whether hNav1.4 mutations causing myotonia may affect blocking effects of mexiletine and influence patients' response to the drug. Indeed, we observed that mutations, like G1306E, shifting the voltage dependence of channel inactivation toward less negative potentials reduce mexiletine effect, likely because of the preferential binding of mexiletine to inactivated channels. In contrast, inhibition of sodium channels by flecainide, another antiarrhythmic drug, was not affected by the mutation. Such observation was successfully translated to humans, since a woman and her son, both carrying the G1306E mutation and suffering from severe myotonia with little response to mexiletine, obtained great improvement by shifting therapy from mexiletine to flecainide [7]. Recently, the same approach was applied to a young girl carrying the P1158L mutation [8]. We are committed to extend this pharmacogenetics approach to a major number of myotonic hNav1.4 mutations, in close collaboration with clinical Neurologists, Parents' associations (“Miotonici in Associazione”), and charities (Italian Telethon Foundation and Association Française contre les Myopathies).

All together, these results open the way toward a precision medicine aimed at identifying the best drug for each mutation or group of similar mutations causing non dystrophic myotonias. Our studies may also serve as a paradigm for other membrane excitability diseases caused by sodium channel mutations, such as cardiac arrhythmia, epilepsy, and chronic pain. Concrete application of precision medicine in rare diseases are already on the market, including CFTR chloride channels ligands for cystic fibrosis or exon-skipping drugs for Duchenne muscular dystrophy. It is likely that such strategies will continue to grow and will allow to better address treatment of rare diseases.
